# Atopic Dermatitis and Comorbidity

**DOI:** 10.3390/healthcare8020070

**Published:** 2020-03-25

**Authors:** Sanja Bekić, Vjenceslav Martinek, Jasminka Talapko, Ljiljana Majnarić, Mila Vasilj Mihaljević, Ivana Škrlec

**Affiliations:** 1 Family Medicine Practice, HR-31000 Osijek, Croatia; sbekic@mefos.hr (S.B.); mvjenceslav@yahoo.com (V.M.); 2 Department of Internal Medicine, Family Medicine and History of Medicine, Faculty of Medicine, Josip Juraj Strossmayer University of Osijek, HR-31000 Osijek, Croatia; ljiljana.majnaric@mefos.hr; 3 Faculty of Dental Medicine and Health, Josip Juraj Strossmayer University of Osijek, HR-31000 Osijek, Croatia; jtalapko@fdmz.hr; 4 Health Center Vukovar, HR-32000 Vukovar, Croatia; milavasilj88@gmail.com

**Keywords:** atopic dermatitis, atopic march, comorbidity, psychological stress

## Abstract

Atopic dermatitis is the most common chronic inflammatory skin disease. It is often the first indicator of allergic diseases, and a certain percentage of patients are affected by allergic rhinitis and/or asthma as a consequence. The study aimed to investigate the link between atopic dermatitis and comorbidity in family medicine. In the specialist family medicine practice Osijek, a retrospective study was conducted in the period from January 1, 2016 to July 1, 2017 on the percentage of patients with atopic dermatitis in the total number of patients, and their comorbid diseases. The data source was the E-chart. The results showed that 195 (10.53%) out of 2056 patients had atopic dermatitis, 80 (41%) patients had atopic dermatitis and allergic rhinitis, 34 (17.4%) asthma, 132 (67.7%) infections, 59 (30.3%) gastrointestinal disturbances, and 68 (34.3%) had mental disorders. Patients up to 18 years old were more likely to have infections, and adult patients were exposed to chronic stress. The most commonly used drug was loratadine (60.5%), while mometasone was the most commonly administered topical drug (40%). The result of this research showed the steps of the ˝atopic march˝. Atopic dermatitis is followed by changes in the skin and its progression to other organ systems in most of the patients.

## 1. Introduction

Atopic dermatitis is the most common chronic relapsing eczema that a family medicine practitioner encounters, compared to other chronic forms of dermatitis. This chronic skin disease, where inflammation is a consequence of barrier dysfunction, is hereditarily determined, most often begins in early childhood and persists, or it does not manifest until adulthood. It is characterized clinically by eczematoid lesions and pruritus [1,2,3]. Its prevalence in children and adults is in a wide range from 1%–20% [4,5,6].

Large variations in prevalence are noticed in countries where there are populations of similar ethnicity, indicating that, alongside genetic predisposition, environmental factors also play a significant role in the pathogenesis of the disease [7,8].

Atopic dermatitis is the first step in the “atopic march” of the formation of allergic diseases, primarily allergic rhinitis and asthma [9,10,11]. It represents the predisposition to recurrent skin infections, whether bacterial or viral, as well as the occurrence of mental disorders, primarily anxiety and depression [12]. Chronic stress leads to exacerbation of the symptoms of allergic dermatitis [13]. In a clinical sense, atopic dermatitis is accepted as a disease, which is at least partially prompted by damage to the surface layer of the skin, which may be acquired or inherited [14]. Through the damaged skin, the immunological system comes into contact with various epicutaneous allergens and microorganisms, and an inadequate immunological response makes these patients subject to bacterial or viral infections [14,15]. Patients with atopic dermatitis have Th1/Th2 dysbalance [16]. The acute phase of atopic dermatitis is dominated by a Th2 profile, while the chronic phase of atopic dermatitis is a more Th1/Th2 mixed pattern. Also, Th22 can be active in the chronic phase [17]. Atopic dermatitis is phenotypically diverse. Highly diverse endotypes of the immune system characterize it. Increased levels of Th17 and Th22 are observed in pediatric atopic dermatitis [18]. Asian atopic dermatitis phenotype is similar to the European phenotype, but has a higher level of Th17, while African American phenotypes had upregulation of Th22 [19].

With the discovery that mutation of the epidermal gene filaggrin predisposes one to the occurrence of allergic dermatitis and asthma, the connection was explained between dermatitis and allergic diseases [20]. The mutation of filaggrin is the greatest risk factor for any disease with a clear genetic foundation, which represents a shift from the previous understanding of atopic diseases as primarily immunological [21]. Filaggrin deficit leads to an increase in the production of thymic stromal lymphopoietin (TSLP), a cytokine which is the link between the skin and the allergic response system [22]. TSLP is created by keratinocytes and promotes the production of Th2 lymphocytes in the damaged skin of patients with atopic dermatitis. Raised levels of TSLP in experimental mice models lead to asthma [23]. The concept of epicutaneous sensitization has been shown experimentally, which indicates the connection between skin damage and an inadequate systemic immunological reaction, which leads to the occurrence of systemic allergic diseases [23].

Filaggrin gene mutations are the most known genetic factor, but numerous environmental and immunological factors affect the manifestation and course of atopic dermatitis. Filaggrin is just one of the genes within the epidermal differentiation complex, and many other genes also contribute to pathogenesis. One such gene encodes hornerin, the level of which is reduced in the skin of patients with atopic dermatitis. The number of transmembrane proteins, such as claudin-1, is also reduced in the skin of patients with atopic dermatitis, thereby increasing the viral penetration [24]. Defective epidermal structural proteins together contribute to the dysfunctional skin barrier underlying atopic dermatitis. Environmental factors can damage structural epidermal proteins, which diminishes skin barrier function and results in increased sensitivity and the onset of atopic dermatitis. Additionally, the damaged skin barrier affects the immune response and the skin microbiome. The complex interaction between the skin barrier and immune activation determines the response to environmental factors, such as allergens and microbes. A change in the composition of the skin microbiome has been observed in patients with atopic dermatitis, which is part of a complex interaction with the skin barrier integrity [19,24].

Highly expressed systemic sensitization to food and airborne allergens may occur, due to the penetration of those allergens through the damaged skin, and also lead to respiratory allergic diseases, such as allergic rhinitis and asthma, and intolerance to certain foods [25].

Acute mental stress exacerbates the symptoms of atopic dermatitis, and often these patients have comorbid depression and anxiety [13,26]. Chronic stress leads to an increase in the number of 5-hydroxytryptamine 2A receptors in the skin, which results in the altered innervation of the skin and the elevated tonus of the sympathetic nervous system, which may also result in the exacerbation of disease symptoms [27].

The primary aim of the study was to evaluate the prevalence of atopic dermatitis and comorbidities. The included comorbidities were allergic (allergic rhinitis and asthma) and non-allergic (infections, gastrointestinal and psychiatric disorders, and metabolic syndrome). The second aim was to examine the topical and systemic treatment of atopic dermatitis.

## 2. Patients and Methods

In the specialized family medicine practice Osijek, retrospective research was undertaken in the period from 1 January 2016 to 1 July 2017, on the proportion of patients with atopic dermatitis and the comorbidities listed, in relation to the total number of patients. All participants signed an informed consent form before inclusion in the study. The study was approved by the Ethical Committee of the Faculty of Medicine Osijek.

The diagnosis of atopic dermatitis is primarily clinically based on the criteria developed by Hanifin and Rajki in 1980, and later revised by the American Academy of Dermatology [28]. There are three basic criteria for diagnosis: itchy eczema, typical morphology and age-specific patterns (lichenification in adults, or facial, neck, and extensor involvement in infants and children), and positive personal and/or family history of atopic diseases (asthma, allergic rhinitis or atopic dermatitis). The additional criteria are chronic or relapsing history of dermatitis, which may occur weakly during active disease. The source of data was the E-chart (e-records).

Infections were mainly respiratory and of the gastrointestinal system. The most common respiratory infections were rhinitis, nasopharyngitis and tonsilitis, of viral or bacterial aetiology, encoded according to the International Classification of Diseases and Related Health Problems, 10th Revision (ICD10) as J00: Acute nasopharyngitis, J01: Acute sinusitis, J02: Acute pharyngitis, J03: Acute tonsilitis, J18: Pneumonia, J20: Acute bronchitis. Infections of the gastrointestinal system related to the diagnosis code A08: Viral and other specified intestinal infections and A09: Infectious gastroenteritis and colitis, unspecified. Gastrointestinal problems presume abdominal pain due to the illnesses listed under K21: Gastro-oesophageal reflux and K29: Gastritis and duodenitis. In the category of mental stress are the illnesses classified under the ICD-10 classification F40: Phobic anxiety disorders, F41: Other anxiety disorders; F43.1 Post-traumatic stress disorder, and F32: Depressive episode. For the treatment of illnesses responsible for mental disorders in patients, benzodiazepines (alprazolam, oxazepam, diazepam), anti-depressives (paroxetine, escitalopram, mirtazapine, venlafaxine) and hypnotics (nitrazepam and zolpidem) are used.

### Statistical Method

The Fi (ϕ) coefficient correlation and McNemar’s χ^2^ test were used to test the statistical significance between the related variables. A P value equal to or less than 0.05 was considered statistically significant. The analysis was conducted using the SPSS software (ver. 16.0, SPSS Inc., Chicago, IL, USA).

## 3. Results

The Fi (ϕ) coefficient correlation was calculated for the calculation of the connections between the dichotomous variables, and McNemar’s χ^2^ test was used for the dependent samples, to test the statistical significance between the related variables.

The percentage was calculated for males and females, and their ages; the subjects were divided into two groups, adults (19 years and older) and children (18 years and less).

Table 1 shows that 195 patients took part in the research, 67 males (34.4%) and 128 females (65.6%), in an age range from 1 to 85 years, with an average age of 42 ± 23 years, for the children group 12 ± 5, and for the adults group 51 ± 19 years.

Of the 195 subjects, 21 (10.77%) had allergic rhinitis and asthma, and 13 (6.67%) had one other associated infection. Mental disturbances together with asthma and allergic rhinitis were recorded in five (2.56%) subjects. Gastrointestinal disturbances and infections were found in 43 (22.05%) subjects, and 25 (12.82%) of them had mental disorders along with gastrointestinal disturbances. Atopic dermatitis along with all these comorbid diseases was recorded in two (1.03%) patients (Table 2). The incidence of atopic dermatitis and comorbid diseases in relation to the patients’ ages are shown in Table 3.

Table 4 shows that the largest number of patients had an infection once (32.3%) or twice (27.2%) and that only one patient had an infection nine times in the period in question.

The most frequently administered perorally drug was loratadine (60.5%), followed in terms of frequency of use by fexofenadine (18.5%). The drug least used was dimetindene (2.5%). The drugs most often used topically were mometasone (32%), betamethasone (22.9%) and betamethasone/gentamicin (18.3%), and betamethasone/salicylic acid was used significantly less. Betamethasone and methylprednisolone were frequently used, diluted in Belobaza cream. Methylprednisolone aceponate and pimecrolimus were used least often, each in only 0.6% of cases (Table 5).

The correlation analysis showed a low, statistically significant, negative correlation between age and infection (r = –0.18), that is, in the children, the incidence of infection is higher. Further, a moderate, statistically significant, positive correlation was found between age and mental stress (r = 0.47), which indicates that adult patients have higher levels of mental stress. Additionally, a low, statistically significant, positive correlation was found between allergic rhinitis and asthma (r = 0.19), that is, patients with allergic rhinitis often also have asthma. In this study, the age of patients positively correlates with cardiovascular diseases (r = 0.619); adult patients more commonly have cardiovascular diseases. Also, the correlation between age and metabolic syndrome is positive (r = 0.292). Mental stress is in positive, statistically significant, correlation with metabolic syndrome and cardiovascular diseases (r = 0.214, and r = 0.249, respectively). A significant positive correlation is found between metabolic syndrome and cardiovascular diseases (r = 0.226) (Table 6).

In order to verify the existence of statistically significant differences between age and infection, age and mental stress, and allergic rhinitis and asthma, McNemar’s tests were used, with the relevant contingency tables. The data from the contingency tables are shown in Table 7, Table 8, and Table 9 for each statistically significant correlation found. The results of the McNemar’s tests are shown jointly in Table 10.

There is a statistically significant difference between the age of the subjects and infection, age and mental stress, and allergic rhinitis and asthma. The differences are significant at the level of *p* < 0.01. It may be concluded that there is a statistically significant connection between the age of the patients and infection and mental stress, that is, children have infections more often, but less often suffer from mental stress, while adult patients less often suffer from infections, but are more often subject to mental stress. A statistically significant connection was found between allergic rhinitis and asthma, which means that patients with allergic rhinitis often have asthma.

## 4. Discussion

Of the total of 2056 patients, 195 (10.54%) had atopic dermatitis, which is in line with the data on the prevalence of atopic dermatitis in the world [7]. Regarding comorbid diseases, allergic rhinitis was found in 41% of subjects, more often in men, while asthma was found in 17.4% of subjects. In this research, 10.77% of the subjects had both allergic rhinitis and asthma. According to the research by Sybilski, the prevalence of allergic rhinitis and asthma in adult patients suffering from atopic dermatitis was 14.6% [29]. Notably, 67.7% of patients suffered from an infection in the period in question, due to a compromised immune system, mainly cell immunity, from damage to the protective surface layer of the skin, and a filaggrin deficit [30]. Infections were more frequent as comorbidities with atopic dermatitis and gastrointestinal disorders (22.05%) than asthma and allergic rhinitis (6.67%). The explanation may perhaps be found in the prevalence of comorbid allergic diseases, which is known to decrease with age. The low incidence of asthma in this study is observed due to the much larger number of adult patients with atopic dermatitis (76.4%) than children (23.6%).

In the present study, the most frequent gastrointestinal problems in children were vomiting, diarrhea, and constipation. Meanwhile, the most common gastrointestinal problems in the adults with atopic dermatitis were irritable bowel disease and gastritis. In this study, children account for only about 23% of the population, with an average age of 12 ± 5 years, and only a few patients with atopic dermatitis likely suffer from concomitant food allergy. The association between atopic dermatitis and food allergy is known to be up to 30% of cases in the first years of life [31].

Yang et al. indicated the presence of mental disorders caused by stress in patients with atopic dermatitis [32]. In this research, 31.9% of patients had mental disorders, in the sense of anxiety and depressive disorders, which were treated with the appropriate medication. According to the literature [33], more than 30% of patients with atopic diseases of the skin have significant psychiatric and psycho-social comorbidities, which is also indicated by the results of this research (34.9% of subjects). Patients who have gastrointestinal disorders as comorbidities more often also have mental disturbances (12.82%), in comparison with the significantly lower number of patients with allergic rhinitis and asthma with associated mental disturbances (2.56%).

A statistically significant correlation was found between the subjects’ ages and infection and mental stress, and between allergic rhinitis and asthma, that is, children suffering from atopic dermatitis were more often subject to infections, and less to mental stress, whilst adult patients more often suffered from the consequences of stress and mental illness than infection [34]. It is well-known that acute respiratory infections are the most frequent illnesses, which mainly occur in children and young patients, and they are mostly treated on the level of primary health care [35]. This may be an explanation for the higher proportion of infections in children in this research. The subjects with comorbid allergic rhinitis more often suffer from asthma, which is well known [11,36].

Metabolic syndrome is likely associated with various dermatological conditions; one of them is atopic dermatitis [37,38]. That combination could contribute to an increased risk of cardiovascular disease in patients with atopic dermatitis [37]. In this study, among 149 adult patients, 17 (8.7%) of them have metabolic syndrome, 13 (6.7%) have diabetes mellitus and 56 (28.7%) have cardiovascular diseases. The incidence of atopic dermatitis was correlated with numerous risk factors, such as triglyceride level, dyslipidemia, obesity, and hypertension [38,39]. The skin is a target organ of insulin-regulating functions in the pathogenesis of inflammatory diseases. Skin disorders should be treated as an initial sign of systemic metabolic diseases [38], and screening for the metabolic syndrome in patients with atopic dermatitis should be considered [39].

The treatment of atopic dermatitis comprises several types of therapies. The primary therapy for barrier dysfunction includes the use of emollients (collodions, creams, gels, lotions, ointments). Topical therapy involves the application of topical corticosteroids and topical calcineurin inhibitors. Systemic therapies comprise immunosuppressants (antihistamines), biologics, and antimicrobial therapy [40,41,42,43,44]. In this research, the most frequently prescribed topical corticosteroid was mometasone (32% of subjects). The second line is topical calcineurin inhibitors, such as tacrolimus and pimecrolimus. In this study, the majority of patients used topical corticosteroids (78%), while 41% of patients used both topical corticosteroids and antihistamines. One patient used a topical calcineurin inhibitor (pimecrolimus) in combination with topical corticosteroids, and antihistamines. The peroral use of antihistamines is recommended to ease itching. The most frequently prescribed antihistamine was loratadine (60.5%). It is believed that histamine H4 receptor antagonists are more effective than H1 antagonists [45]. The topical use of antihistamines in the treatment of atopic dermatitis is not recommended. Some patients benefit from phototherapy, and UV light can reduce the symptoms of pruritus [43,46,47,48].

The Global Burden of Disease project has revealed that skin diseases are the fourth leading cause of non-fatal disease burden globally. Dermatitis had the most significant burden of skin conditions. Skin conditions represent a significant threat to patients’ well-being, mental health, ability to function, and social inclusion. Skin diseases influence a person’s ability to be involved and engaged in relations with others [49].

There are some limitations in this study. A shortcoming of this research was the deficiency of the records on skin infections comorbid with atopic dermatitis, so that information could not be taken into account in this research, and isolation and proof of the microbial pathogens of those diseases are extremely rare. Another weakness of this research is the deficiency in the records of food allergies, as a well-known cause of gastrointestinal disorders in comorbidity with atopic dermatitis. However, in this study, children represent only about 20% of the population, and only a few patients with atopic dermatitis likely suffer from concomitant food allergy. Further, also due to the deficiency of the information from the medical documents, there was no information on the atopic course from childhood to adulthood. It was not possible to discern a causal relationship between mental disturbances and skin changes, due to the incomplete records in the medical documentation, in the sense of the time when those illnesses occurred. There is also no information about the existence of disorders in the neurophysiological development of the child, and disorders with a possible genetic basis, which exist in patients suffering from atopic dermatitis [13,36]. These disorders may have led in later life to mental disturbances, but mental disturbances may also have led to skin changes, in the sense of atopic dermatitis. Despite the small number of subjects and the fact that this research was conducted in a single practice, the results showing the prevalence of atopic dermatitis and comorbid diseases and their inter-relationship are in line with the data given in the literature consulted.

## 5. Conclusions

The main results of the present study are that children had a higher prevalence of infections, while adult patients had a higher prevalence of mental disorders. According to statements from the literature, as well as the results of this research, the beginnings of atopic disease changes on the skin may be clearly determined, as well as its progression to other organ systems. This is known as the “atopic march”. Progression to the respiratory system prompts the occurrence of allergic rhinitis and asthma. Mental disorders are also recorded in these patients. All these disorders have an altered cellular immune system in their pathogenesis, with a genetic predisposition for their occurrence, both of atopic dermatitis and comorbid diseases.

## Figures and Tables

**Table 1:** The incidence and descriptive data of patients with atopic dermatitis and comorbidities.

Variable		Frequency Total	% Total
**Sex**	M	67	34.4
	F	128	65.6
Age	Children	46	23.6
	Adults	149	76.4
Atopic dermatitis		195	100
Allergic rhinitis		80	41
Asthma		34	17.4
Infections		132	67.7
Gastrointestinal problems		59	30.3
Mental stress/therapy used		68	34.9
Metabolic syndrome		17	8.7
Diabetes mellitus		13	6.7
Cardiovascular diseases		56	28.7

M = men; F = women.

**Table 2:** The incidence of atopic dermatitis and comorbidities in relation to the sex of the patients.

**Atopic Dermatitis**	**Sex**		**Total**
	**M**	**F**	
**N** %	67	128	195
	100	100	100
Allergic rhinitis	Sex		Total
	M	F	
N %	32	48	80
	32	37.5	41
Asthma	Sex		Total
	M	F	
N %	12	22	34
	12	17.2	17.4
Infections	Sex		Total
	M	F	
N %	47	85	132
	47	66.4	67.7
Gastrointestinal problems	Sex		Total
	M	F	
N %	16	43	59
	16	33.6	30.3
Mental stress/therapy used	Sex		Total
	M	F	
N %	21	47	68
	21	36.7	34.9

M = male; F = female; N = number of subjects.

**Table 3:** The incidence of atopic dermatitis and comorbid diseases in relation to the patients’ ages.

**Atopic Dermatitis**	**Age**		**Total**
	**Children**	**Adults**	
N %	46	149	195
	23.6	76.4	100
Allergic rhinitis	Age		Total
	Children	Adults	
N %	22	58	80
	47.8	38.9	41
Asthma	Age		Total
	Children	Adults	
N %	13	21	34
	28.3	14.1	17.4
Infections	Age		Total
	Children	Adults	
N %	39	93	132
	84.7	62.4	67.7
Gastrointestinal problems	Age		Total
	Children	Adults	
N %	11	48	59
	23.9	32.2	30.2
Mental stress/therapy used	Age		Total
	Children	Adults	
N %	3	65	68
	6.5	43.6	34.8

N = the number of subjects.

**Table 4:** Distribution of patients according to the number of infections.

The Number of Infections	The Number of Patients	% of Patients
1	63	32.3
2	53	27.2
3	32	16.4
4	26	13.3
5	8	4.1
6	4	2.1
7	3	1.5
8	5	2.6
9	1	0.5
Total	195	100

**Table 5:** The proportion of medications administered most often, per os or topically.

Routes of Drug Administration	Type of Medication	Frequency	%
Per os	Loratadine	49	60.5
	Fexofenadine	15	18.5
	Desloratadine	9	11.1
	Bilastine	6	7.4
	Dimetindene	2	2.5
Total per os		81	100
Topically	Mometasone	49	32
	Betamethasone	35	22.9
	Betamethasone/gentamicine	28	18.3
	Betamethasone/salicylic acid	20	13.1
	Betamethasone 20%	5	3.3
	Alclometasone	4	2.6
	Betamethasone/clotrimazolum/gentamicine	3	2
	Dexamethasone	2	1.3
	Betamethasone 40%	2	1.3
	Mometasone 40%	1	0.6
	Betamethasone 60%	1	0.6
	Methylprednisolone	1	0.6
	Methylprednisolone aceponate	1	0.6
	Pimecrolimus	1	0.6
Total topically		153	100

**Table 6:** Correlation analysis of the variables of sex, age, and comorbid diseases in patients with atopic dermatitis.

Variable	1	2	3	4	5	6	7	8	9	10
1. Sex	–	−0.17	0.10	0.10	0.04	−0.10	−0.05	0.045	0.107	0.197**
2. Age		–	−0.05	−0.11	–0.18*	0.12	0.47**	0.292**	0.165*	0.619**
3. Allergic rhinitis			–	0.19**	0.11	0.02	−0.09	–0.158*	−0.014	0.001
4. Asthma				–	0.03	0.05	0.08	−0.100	−0.069	0.007
5. Infections					–	0.07	−0.07	–0.147*	−0.062	–0.224**
6. Gastrointestinal disorders						–	−0.10	0.020	0.137	0.075
7. Mental stress							–	0.214**	−0.109	0.249**
8. Metabolic syndrome								–	0.127	0.226**
9. Diabetes mellitus									–	0.148*
10. Cardiovascular diseases										–

The bolded correlation coefficients are statistically significant at * *p* < 0.05 or ** *p* < 0.01.

**Table 7:** Contingency table of correlation of age and infection.

**Age**		**Infections**		**Total**
		**Negative**	**Positive**	
Children	Noted	7	39	46
	Theoretical	14.5	31.5	46
Adults	Noted	56	93	149
	Theoretical	48.5	100.5	149
Total	Noted	63	132	195
	Theoretical	63	132	195

**Table 8:** Contingency table of correlation between age and mental stress.

**Age**		**Mental stress**		**Total**
		**Negative**	**Positive**	
Children	Noted	43	3	46
	Theoretical	30.2	15.8	46
Adults	Noted	84	65	149
	Theoretical	86.8	52.2	149
Total	Noted	127	68	195
	Theoretical	127	68	195

**Table 9:** Contingency table of the correlation between allergic rhinitis and asthma.

**Allergic rhinitis**		**Asthma**		**Total**
		**Negative**	**Positive**	
**None**	**Noted**	102	13	115
	Theoretical	94.9	20.1	115
Present	Noted	59	21	80
	Theoretical	66.1	13.9	80
Total	Noted	161	34	195
	Theoretical	161	34	195

**Table 10:** McNemar’s χ^2^ quadratic test for dependent samples, for testing the significance of differences between the variables age*infection, age*mental stress and allergic rhinitis*asthma.

Statistics	Age*infection	Age*mental stress	Allergic rhinitis*asthma
N	195	195	195
McNemar’s χ2	9.553	15.574	28.125
*p*–value	0.002	<0.0001	<0.0001

N = number of subjects; p = statistical significance (*p* < 0.05).
